# Kank1 Is Essential for Myogenic Differentiation by Regulating Actin Remodeling and Cell Proliferation in C2C12 Progenitor Cells

**DOI:** 10.3390/cells11132030

**Published:** 2022-06-26

**Authors:** Mai Thi Nguyen, Wan Lee

**Affiliations:** 1 Department of Biochemistry, Dongguk University College of Medicine, 123 Dongdae-ro, Gyeongju 38066, Korea; nguyenmainhp@gmail.com; 2 Channelopathy Research Center, Dongguk University College of Medicine, 32 Dongguk-ro, Ilsan Dong-gu, Gyeonggi-do, Goyang 10326, Korea

**Keywords:** Kank1, actin filament, myogenesis, differentiation, proliferation

## Abstract

Actin cytoskeleton dynamics are essential regulatory processes in muscle development, growth, and regeneration due to their modulation of mechanotransduction, cell proliferation, differentiation, and morphological changes. Although the KN motif and ankyrin repeat domain-containing protein 1 (Kank1) plays a significant role in cell adhesion dynamics, actin polymerization, and cell proliferation in various cells, the functional significance of Kank1 during the myogenic differentiation of progenitor cells has not been explored. Here, we report that Kank1 acts as a critical regulator of the proliferation and differentiation of muscle progenitor cells. Kank1 was found to be expressed at a relatively high level in C2C12 myoblasts, and its expression was modulated during the differentiation. Depletion of Kank1 by siRNA (siKank1) increased the accumulation of filamentous actin (F-actin). Furthermore, it facilitated the nuclear localization of Yes-associated protein 1 (YAP1) by diminishing YAP1 phosphorylation in the cytoplasm, which activated the transcriptions of YAP1 target genes and promoted proliferation and cell cycle progression in myoblasts. Notably, depletion of Kank1 suppressed the protein expression of myogenic regulatory factors (i.e., MyoD and MyoG) and dramatically inhibited myoblast differentiation and myotube formation. Our results show that Kank1 is an essential regulator of actin dynamics, YAP1 activation, and cell proliferation and that its depletion impairs the myogenic differentiation of progenitor cells by promoting myoblast proliferation triggered by the F-actin-induced nuclear translocation of YAP1.

## 1. Introduction

Skeletal muscle is essential for a diversity of body functions, including locomotor activity, postural behavior, respiration, and metabolic homeostasis [1]. Skeletal myogenesis is a tightly regulated sequential cellular process involving satellite cell activation, cell cycle exit, myogenic gene expression, differentiation, and myotube formation, and it is crucially required for muscle development, regeneration, and growth [2]. Hence, dysregulation of myogenesis is inextricably linked to muscle wasting, which is characterized by loss of muscle mass, quality, strength, and regeneration ability [3]. Interestingly, recent studies have unveiled that actin cytoskeleton dynamics, in concert with molecules that regulate actin remodeling modulate Hippo signaling, cell proliferation, the cell cycle, and cell differentiation, play essential roles during skeletal myogenesis [4,5]. However, the molecular identities and mechanisms underlying actin dynamics and myogenesis remain largely unknown.

Actin is a highly conserved major cytoskeletal component and primarily drives cell motility and shape alterations by regulating the dynamic assembly and disassembly of filamentous actin (F-actin) [6,7]. Actin cytoskeleton dynamics are also involved in various cellular functions, such as the modulation of cell proliferation, the cell cycle, and multiple signal transduction mechanisms [8,9]. Moreover, studies from the last decade have demonstrated that actin dynamics are closely related to cellular mechanotransduction and progenitor cell differentiation in skeletal muscle [2,6]. Mechanistically, increased actin polymerization is implicated in the activation of Yes-associated protein 1 (YAP1) through the suppression of the Hippo signaling pathway, which is required for modulations of tissue and organ sizes [4,5]. YAP1 is a transcriptional co-activator that regulates a broad range of biological processes, including survival, proliferation, migration, and differentiation [10]. It has been suggested that the dysregulation of actin dynamics and accumulation of F-actin trigger mechanotransduction response, provoke the nuclear localization of YAP1, and consequently stimulate cell proliferation [11]. Furthermore, many molecules that participate in actin cytoskeleton dynamics between the actin monomer and F-actin have been found to be critically required for myogenesis [12,13,14].

Kank1 is a member of the KN motif and ankyrin repeat domain-containing protein family, members of which are expressed ubiquitously in mammalian tissues as integrin adhesion complex components [15,16,17,18]. The Kank family proteins (Kank1–4) have been suggested to regulate adhesion dynamics by coordinating force transmission and recruiting the microtubules for focal adhesion [19]. In the cytoplasm, Kank1 has been reported to negatively regulate actin polymerization and thus disassemble F-actin, which controls cytoskeleton formation [20,21]. Overexpression of Kank1, therefore, was found to inhibit F-actin formation induced by Wnt3a in NIH3T3 cells [22]. Kank1 is also known to play a significant role in the inhibition of cell proliferation by arresting the cell cycle and promoting proapoptotic pathways [23,24,25,26]. The Kank1 gene was first identified to act as a tumor suppressor in kidney cancer [23], and the induction of Kank1 has been found to inhibit cell proliferation [23,24] and promote the mitochondrial apoptotic signaling cascade [25,26]. Furthermore, its downregulation was subsequently reported in several malignancies, including gastric cancer [25], oral squamous carcinoma [26], colorectal carcinoma [27], and breast cancer [28]. Although it has been established that Kank1 is involved in actin cytoskeleton dynamics and cell proliferation, which are involved in myogenesis, its functional significance in the differentiation of myogenic progenitor cells remains to be explored.

In this study, we hypothesized that Kank1 could be modulated during the myogenic differentiation of progenitor cells and that its downregulation might enhance F-actin formation and cell proliferation and be causally linked to impaired myogenic differentiation. In this regard, we investigated the expression of Kank1 and its relation to the expression of myogenic genes in C2C12 myoblasts. Furthermore, we analyzed the effects of Kank1 depletion on YAP1 activation and cell proliferation and explored the effects of Kank1 on myogenic factor activation and myoblast differentiation.

## 2. Materials and Methods

### 2.1. Cell Culture and Differentiation

C2C12 cells, an immortalized murine myogenic progenitor cell line (ATCC, Manassas, VA, USA), were cultured in a growth medium (GM, DMEM (Gibco, Carlsbad, CA, USA) containing 10% fetal bovine serum (FBS) and 1% penicillin/streptomycin) using a 37 °C incubator under 5% CO_2_. To differentiate C2C12 myoblasts into myotubes, the GM was replaced with a differentiation medium (DM, DMEM containing 2% horse serum (Gibco) and 1% penicillin/streptomycin) when cell density reached 80–90% confluence. The medium was changed daily. Unless otherwise stated, all materials and reagents were obtained from Sigma-Aldrich (St. Louis, MO, USA).

### 2.2. Transfection of Oligonucleotides

C2C12 cells (1.3 × 10^5^ cells) were seeded in 35 mm dishes 24 h before transfection and then transfected with a scrambled control RNA (scRNA) or Kank1 siRNA (siKank1)(Genolution, Seoul, Korea) in free DMEM without FBS using Lipofectamine 2000 (Invitrogen, Carlsbad, CA, USA), following the manufacturer’s instruction. After 4 h, the medium was replaced with a fresh GM. The sequences of the oligonucleotides used in this study are shown in Table S1.

### 2.3. RNA Extraction and Quantitative Real-Time PCR (qRT-PCR)

After 24 h of transfection, total RNA was purified using the RNeasy Mini Kit (Qiagen, Hilden, Germany) and then subjected to RNase-free DNase digestion, according to the manufacturer’s instructions. Complementary DNA (cDNA) was synthesized using a miScript II RT Kit (Qiagen, Hilden, Germany) from total cellular RNA (1 μg). *q*RT-PCR was applied to determine the relative expression levels of mRNAs using specific primers, SYBR Green I and iTaq polymerase (Promega, Medison, WI, USA), and Light-Cycler 480 software (Roche Applied Science, Penzberg, Germany). The primers and reaction conditions used for *q*RT-PCR are listed in Table S2. We calculated the relative expression levels between groups using the comparative delta CT method (2^−ΔΔCt^) with a control sample (GAPDH) as the reference point.

### 2.4. Preparation of the Cytoplasmic and Nuclear Fractions

Cytoplasmic and nuclear fractions were prepared using the NE-PER^TM^ Nuclear and Cytoplasmic Extraction Reagents (Thermo Fisher Scientific). Briefly, 24 h after transfection, C2C12 cells were harvested using trypsin EDTA and centrifuged at 3000 rpm for 5 min at 4 °C. The pellets were incubated with 100 µL CER I for 30 min and 5.5 µL CER II for 1 min. After centrifugation, the supernatants (cytoplasm extract) were collected into tubes. The insoluble fraction containing nuclei was purified with 50 µL ice-cold NER solution, then centrifuged at 3000 rpm for 10 min at 4 °C. Cytoplasm and nuclear-lysate proteins were prepared as immunoblots described above.

### 2.5. Immunoblot Analysis

The total protein from the transfected cells was obtained using a lysis buffer containing 1% phosphatase inhibitor cocktail II, 0.2 mM PMSF, and 2% Trixon-X (Sigma-Aldrich, St. Louis, MO, USA), and protein concentration was determined using a Bradford assay kit (Bio-Rad, Hercules, CA, USA). Protein samples (20 µg) were resolved on SDS-PAGE gels and transferred to nitrocellulose membranes (Amersham, Braunschweig, Germany), which were blocked with 5% skim milk in TBS-Tween 20 (TTBS) for 1 h and then incubated overnight with the indicated primary antibodies (Table S3) at 4 °C, followed by incubation with secondary antibodies (Table S3) for 1 h at room temperature. Blots were developed using a Femto reagent (Thermo Fisher Scientific, Waltham, MA, USA) with a Fusion Solo Chemiluminescence Imaging System (Vilber Lourmat, Marne-la-Vallée, France), and their densities were analyzed with Evolution Capt software (Vilber Lourmat). β-Actin protein levels were used for normalization.

### 2.6. Immunofluorescence Analysis

After transfection with the oligonucleotides (Table S1), C2C12 myoblasts were differentiated for five days. The myotubes were fixed with formaldehyde-phosphate buffer (4%, 10 min), permeated with Triton X-100 (0.3%, 15 min), and blocked with bovine serum albumin (0.3%, 2 h) at room temperature. Cells were then incubated with myosin heavy chain (MyHC) antibody (1:100; DSHB, Iowa City, IA, USA) at 4 °C overnight, washed three times with PBS, and then incubated for an additional 1.5 h with fluorescently labeled Alexa Fluor 488 (1:200; Invitrogen, Waltham, MA, USA) for 1.5 h. For F-actin staining, cells were fixed and incubated for 40 min with 50 μg/mL FITC-coupled phalloidin (P5282, Sigma, USA), as described previously [14]. Hoechst 33342 (Invitrogen) was used as a nuclear counterstain. The images of MyHC stained cells were captured using a Leica microscope (Leica Microsystems, Mannheim, Germany) and analyzed using ImageJ software (NIH). Differentiation indices were calculated by expressing the numbers of MyHC-positive myotubes containing nuclei as percentages of total nuclei. Fusion indices were calculated by dividing the number of elongated myotube structures containing three or more nuclei by the total number of nuclei. Average widths of myotubes were determined using more than 20 measurements. All measurements were obtained using five images from at least three independent experiments.

### 2.7. Cell Proliferation Assays

Cell proliferation was analyzed using the Click-iT™ EdU Cell Proliferation Assay Kit (Invitrogen, Waltham, MA, USA). Cells (3 × 10^4^ cells) were plated on 8-well chamber slides and transfected for 24 h before measurements. Briefly, cells were incubated in a 250 μL GM containing EdU (10 μM) for 4 h at 37 °C. Cells were then fixed, permeabilized, and incubated with a 250 μL Click-iT reaction cocktail for 20 min. Nuclei were counterstained with Hoechst 33,342 for 15 min. EdU-stained C2C12 cells were observed, and images (five randomly selected fields/samples) were taken using a Leica microscope. Percentages of EdU-positive cells versus total cells were calculated using ImageJ software. We conducted every experiment at least three times.

### 2.8. Cell Viability Analysis

C2C12 cells were seeded into 96-well plates at 10^3^ cells/well and transiently transfected with the oligonucleotides using Lipofectamine 2000 (Invitrogen). The next day, 100 μL of GM containing 10 μL of Quanti-max^TM^ WST-8 Cell Viability Assay Kit reagent (BioMax, Seoul, Korea) was dispensed to each well and cultured for 4 h at 37 °C. Absorbances at 450 nm were measured using a microplate reader (Model 680, Bio-Rad).

### 2.9. Flow Cytometry

Cells were harvested 24 h after transfection using trypsin EDTA (Gibco) and centrifuged at 3000 rpm for 5 min at 4 °C. Pellets were fixed with 70% ethanol overnight at 4 °C, rinsed with PBS buffer, centrifuged, and retrieved. After discarding supernatants, cells were treated with 500 µL of a Cell Cycle kit reagent (C03551; Beckman Coulter, Miami, FL, USA) and incubated for 20 min in the dark. Detection was performed using a CytoFLEX unit (Beckman Coulter).

### 2.10. Statistical Analysis

All data were obtained from at least three independent experiments. Results are expressed as means ± SEMs, and the statistical analysis was performed using Student’s *t*-test for unpaired data.

## 3. Results

### 3.1. Kank1 Expression Was Altered during Myoblast Differentiation

**Figure 1:**
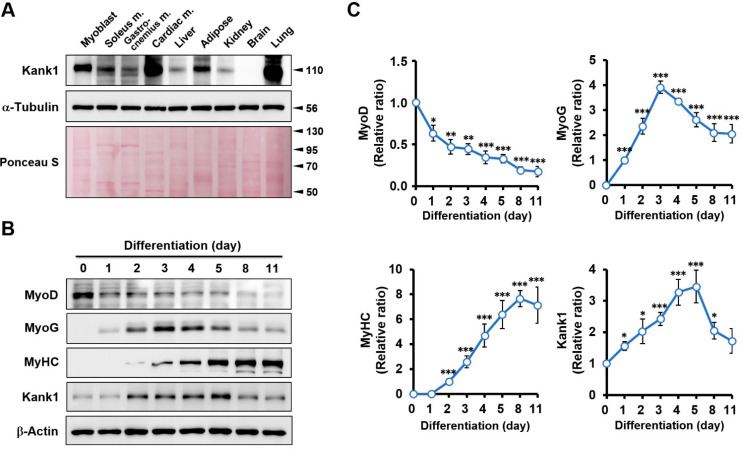
Expression profiles of Kank1 in murine tissues and myogenic differentiation. (**A**) Equal amounts (20 μg/lane) of protein were prepared from C2C12 myoblasts and adult male mouse tissues and subjected to SDS-PAGE (10%) and immunoblot analysis for Kank1. α-Tubulin was used as the loading control. Representative images are shown. (**B**) C2C12 cells were allowed to differentiate for 11 days. The expression of MyoD, MyoG, MyHC, and Kank1 was determined by immunoblotting. β-Actin was used as the loading control. (**C**) Expression levels in immunoblots were determined by densitometry and normalized versus ß-actin. Values are expressed as the relative ratio, with the intensity of differentiation on day 0 (MyoD and Kank1), day 1 (MyoG), or day 2 (MyHC) set to one. All results are presented as means ± SEMs (*n* > 3), and levels of significance are presented as *, *p* < 0.05; **, *p* < 0.01; ***, *p* < 0.001 vs. differentiation day 0.

To investigate the significance of Kank1 in myogenic differentiation, the protein expression of Kank1 was analyzed in various murine tissues, such as myoblasts, skeletal muscle, cardiac muscle, liver, and lung (Figure 1A). Kank1 was widely distributed in most tissues, with relatively high levels in myoblasts, cardiac muscle, and the lungs. We also found relatively low expressions of Kank1 in non-muscle tissues, including the liver and kidney. Although Kank1 was expressed at a moderate level in skeletal muscles (i.e., soleus and gastrocnemius muscles), the expression level of Kank1 in C2C12 myoblasts, which are widely used as a skeletal muscle progenitor cell line, was found to be much higher than in skeletal muscle tissues. This result is consistent with the Human Protein Atlas, which states that Kank1 is a protein expressed ubiquitously and enriched in skeletal and cardiac muscle [29]. Therefore, we next evaluated the modulation of Kank1 expression during the differentiation of myogenic progenitor cells. C2C12 myoblasts were differentiated for up to 11 days in DM, and the expression levels of Kank1, myogenic regulatory factors, and a terminal differentiation marker MyHC were analyzed (Figure 1B,C). Using our differentiation protocol, the protein expression of myoblast determination protein 1 (MyoD), the earliest transcription factor for myogenic commitment, was highly expressed on the initiation of differentiation and gradually diminished. On the other hand, the expression level of myogenin (MyoG, a transcription factor that promotes the expressions of muscle-specific target genes) significantly increased from day 1 to day 3 of differentiation and then markedly declined after day 3. In addition, the expression of MyHC was detectable from day 2 of differentiation and sustained, increasing gradually and peaking at day 5. Interestingly, Kank1 protein levels were regulated similarly to MyoG levels during myoblast differentiation (Figure 1B,C); Kank1 increased during the first four days and declined after day 5 of differentiation. Therefore, we hypothesized that Kank1 might be implicated in myogenic differentiation during skeletal myogenesis.

### 3.2. Depletion of Kank1 Promoted the Nuclear Translocation of YAP1

**Figure 2:**
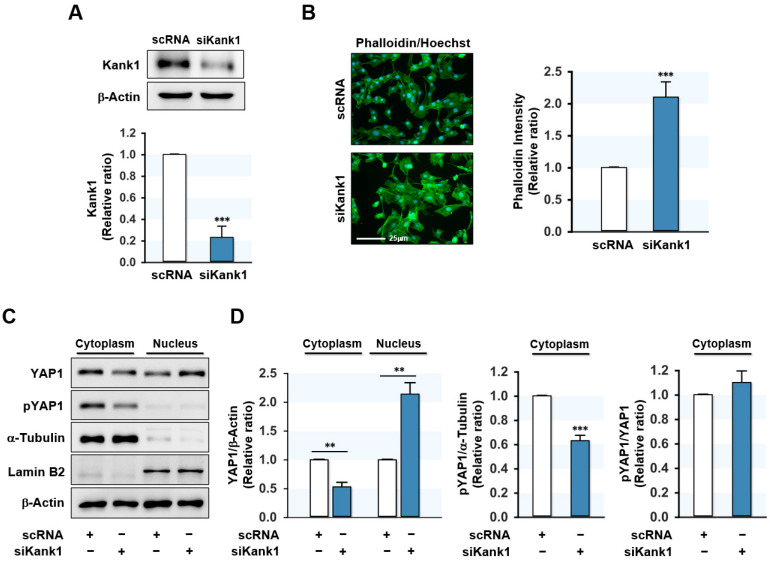
Depletion of Kank1 increased F-actin and nuclear YAP1. C2C12 cells were transfected with control (scRNA) or Kank1 siRNA (siKank1) at a concentration of 200 nM for 24 h. (**A**) Kank1 expressions were analyzed by immunoblotting and then intensities of densitometry were normalized versus ß-actin. Values are expressed as relative ratios versus scRNA control. (**B**) After transfection, cells were stained with FITC-phalloidin (green) and Hoechst 33,342 (blue) for the F-actin and nucleus, respectively. Scale bar: 25 μm. Phalloidin intensities were determined with ImageJ software. (**C**,**D**) The expressions of cytoplasmic and nuclear YAP1 and phosphorylated YAP1 (pYAP1) were analyzed by immunoblotting. The quality of subcellular fractions was verified with cytoplasmic (α-tubulin) or nuclear (lamin B2) markers. Values are shown as relative ratios versus scRNA control. All results are presented as means ± SEMs (*n* > 3), and levels of significance are presented as **, *p* < 0.01; ***, *p* < 0.001 vs. scRNA controls.

Since Kank1 is involved in the negative control of actin polymerization [20,21], Kank1 depletion might augment F-actin levels in myogenic progenitor cells. C2C12 myoblasts were transfected with scRNA or Kank1 siRNA (siKank1), and then F-actin levels were assessed by FITC-coupled phalloidin binding. Transfection with siKank1 reduced the Kank1 protein level by ~75% compared to scRNA in C2C12 myoblasts (Figure 2A), and F-actin level was dramatically enhanced by siKank1 (Figure 2B). The augmented F-actin level was considered to be due to insufficient Kank1 expression, as cellular levels of actin remained steady during the entire differentiation stage.

It has been suggested that F-actin accumulation promotes the nuclear localization of YAP1 by reducing its phosphorylation, consequently activating genes involved in cell proliferation [30]. When we investigated whether Kank1 knockdown affects the phosphorylation and cellular localization of YAP1, we found that siKank1 transfection markedly diminished YAP1 phosphorylation in the cytoplasm of myoblasts and elevated the level of YAP1 in the nucleus (Figure 2C,D). The pYap1/YAP ratio in the cytoplasm did not change from siKank1 transfection, indicating that decreased phosphorylation of YAP1 by siKank1 correlated with reduced YAP1 in the cytoplasm. These results suggest that Kank1 depletion caused the F-actin-induced nuclear translocation of YAP1.

### 3.3. Kank1 Knockdown Induced Myoblast Proliferation

**Figure 3:**
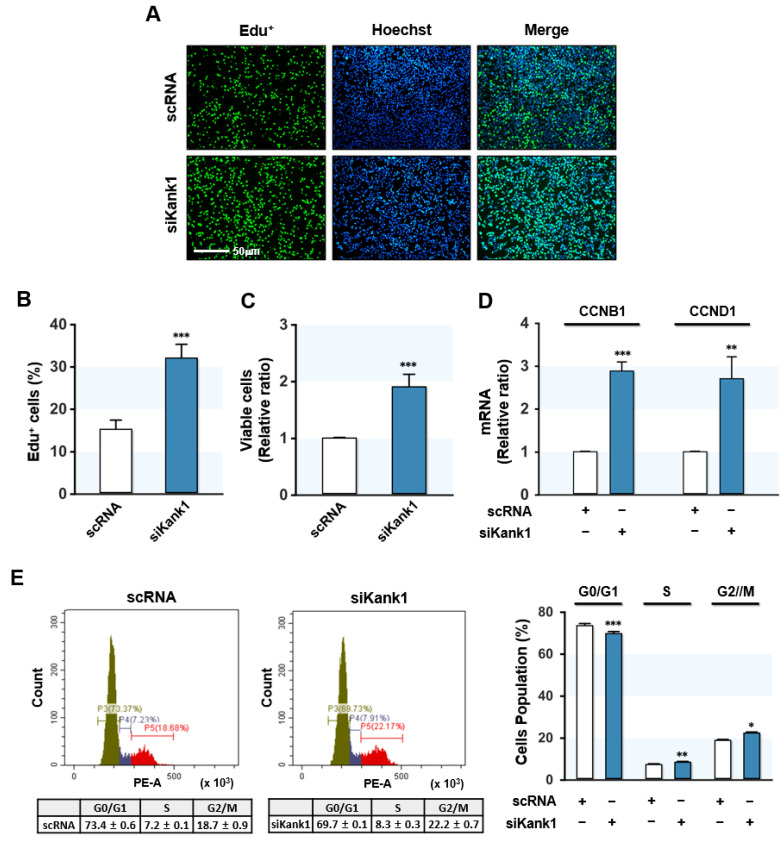
Kank1 knockdown promoted myoblast proliferation and cell cycle progression. C2C12 cells were transfected with control (scRNA) or Kank1 siRNA (siKank1) for 24 h. (**A**) The cells undergoing DNA replication were labeled with EdU (green), and nuclei were stained with Hoechst 33,342 (blue). Scale bar, 50 µm. **(B)** Percentages of EdU-positive cells were determined using ImageJ software. (**C**) Viable cells were quantified using a cell viability assay kit. (**D**) The mRNA levels of CCNB1 and CCND1 were analyzed by *q*RT-PCR and normalized versus GAPDH. (**E**) Flow cytometry was used with a scatter plot for cell cycle analysis of myoblasts at 24 h after transfection. The graph represents one of five independent experiments. All results are presented as means ± SEMs (*n* > 3), and levels of significance are presented as *, *p* < 0.05; **, *p* < 0.01; ***, *p* < 0.001 vs. scRNA controls.

We next analyzed whether Kank1 depletion stimulates proliferation and cell cycle progression in myoblasts, since Kank1 knockdown increased nuclear YAP1 (Figure 2). C2C12 cell proliferation was assessed through EdU incorporation analysis and a cell viability assay 24 h after transfection with scRNA or siKank1. Based on EdU incorporation analysis, siKank1 significantly enhanced the percentage of EdU-positive myoblasts by 2-fold (Figure 3A,B). Similarly, the cell viability assay revealed that siKank1 enhanced the number of viable cells (Figure 3B). These results suggested that depletion of Kank1 promoted myoblast proliferation.

Next, we investigated whether Kank1 knockdown affected the expression of YAP1 target genes involved in the cell cycle progression. As shown in Figure 3C, the *q*RT-PCR analysis revealed that Kank1 depletion caused a significant upregulation of the mRNA levels of CCNB1 and CCND1. In addition, FACS-based cell cycle analysis exhibited enhanced cell cycle progression as a result of siKank1. More specifically, Kank1 knockdown decreased the percentage of cells in the G0/G1 phase, whereas it increased the percentage in the S and G2/M phases (Figure 3D). Collectively, depletion of Kank1 upregulated the expression of YAP1 target genes and facilitated cell proliferation and cell cycle in C2C12 myoblasts.

### 3.4. Kank1 Knockdown Suppressed Myogenic Differentiation

**Figure 4:**
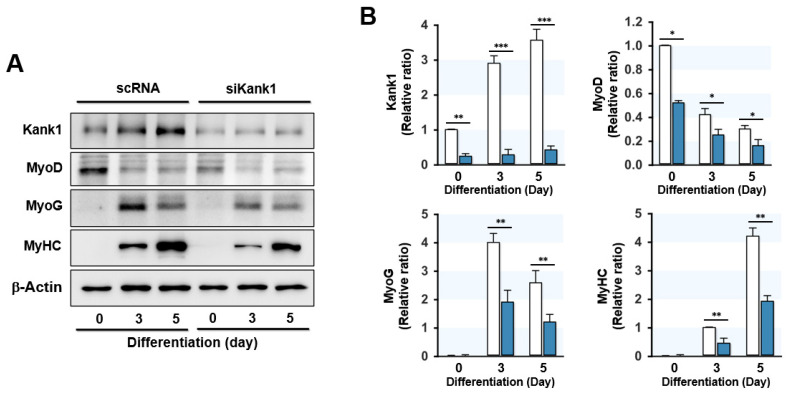
Depletion of Kank1 inhibited myogenic regulatory factor expression. C2C12 cells were transfected with control (scRNA) or Kank1 siRNA (siKank1) and differentiated for 5 days. (**A**) Representative immunoblots of Kank1, MyoD, MyoG, and MyHC on differentiation days 0, 3, and 5. (**B**) The band intensities of protein expressions were quantified using Evolution Capt software and normalized to ß-actin. All results are presented as means ± SEMs (*n* > 3), and levels of significance are presented as *, *p* < 0.05; **, *p* < 0.01; ***, *p* < 0.001 vs. scRNA controls.

Since myoblast proliferation inhibits myogenic differentiation [2], we investigated whether Kank1 knockdown impairs the expression of myogenic regulatory factors, differentiation, and myotube formation in progenitor cells. C2C12 cells were transfected with scRNA or siKank1, and myogenic factor expressions and myotube formation were determined on days 3 and 5 of differentiation, respectively. Depletion of Kank1 by siKank1 markedly suppressed the protein expression of myogenic regulatory factors MyoD and MyoG (Figure 4A,B), and as a result, MyHC expression in siKank1-transfected cells was dramatically lower than in the scRNA control (Figure 4A,B). This observation suggests that Kank1 is essential for the expression of myogenic transcription factors and the differentiation of myoblasts.

**Figure 5:**
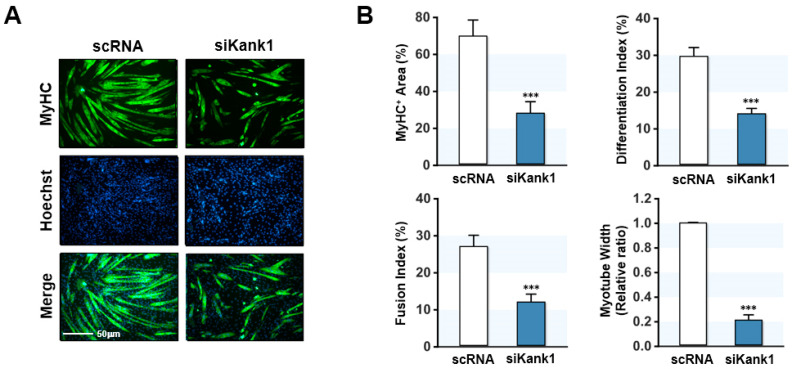
Kank1 knockdown impeded myogenic differentiation and myotube formation. C2C12 cells were differentiated for 5 days after transfection with control (scRNA) or Kank1 siRNA (siKank1). (**A**) Representative immunocytochemistry stained with MyHC antibody (green) and Hoechst 33,342 (blue). Scale bar: 50 μm. (**B**) MyHC-positive areas, differentiation indices, fusion indices, and myotube widths were determined as described in the Materials and Methods section. All results are presented as means ± SEMs (*n* > 3), and levels of significance are presented as ***, *p* < 0.001 vs. scRNA controls.

Furthermore, immunocytochemistry on differentiation day 5 showed that Kank1 knockdown markedly suppressed myotube development (Figure 5A). Moreover, analysis of percentage areas of MyHC-positive cells, differentiation indices, fusion indices, and myotube widths demonstrated that Kank1 depletion inhibited myogenic differentiation and myotube formation (Figure 5B). Overall, these results demonstrate that Kank1 is a crucial regulator of myogenic differentiation and myotube formation in C2C12 cells.

## 4. Discussion

Although Kank1 is involved in the negative regulation of actin polymerization [20,31,32] and actin remodeling is crucial for myogenesis [2,6,8], the functional significance of Kank1 in the myogenic differentiation of progenitor cells has not been previously explored. In this study, we deduced that Kank1 is essential for myogenic differentiation for the following reasons: (i) The expression of Kank1 was modulated during the differentiation of C2C12 myoblasts; (ii) Kank1 knockdown by siRNA significantly increased F-actin and nuclear YAP1 levels in myoblasts; (iii) Kank1 knockdown also enhanced cell cycle progression and myoblast proliferation; and (iv) Kank1 knockdown drastically inhibited the expressions of myogenic regulatory factors and thus hindered differentiation of myoblast. Hence, our study shows that Kank1 is a crucial regulatory player in F-actin-induced YAP1 activation, cell proliferation, and myogenic differentiation.

We reported for the first time that the expression of Kank1 is modulated during myoblast differentiation and myotube formation. Based on immunoblots in C2C12 cells, Kank1 expression was upregulated during the early stage of differentiation and subsequently declined to the basal level after myotube formation, as was observed for MyoG expression (Figure 1). This result implies that Kank1 expression is either required for myogenesis or, at the least, regulated by the differentiation process of myoblasts. Notably, the key findings of our study are that Kank1 depletion markedly stimulated cell proliferation and subsequently inhibited myoblast differentiation. These findings are interesting because the proliferation and myogenic differentiation of progenitor cells are regulated inversely throughout myogenesis, and the inhibition of progenitor cell proliferation with cell cycle arrest is necessary for myoblast differentiation and myotube formation [2,33]. In this regard, the inhibition of myoblast differentiation by Kank1 depletion appeared to be attributable to the promotion of myoblast proliferation and cell cycle progression before the inhibition of myogenic regulatory factor expression. Recent studies on the roles of Kank1 support an inverse relationship between Kank1 expression and cell proliferation in different cell types. Kank1 was initially reported to suppress the growth of renal cell carcinomas by arresting the cell cycle in the G_0_/G_1_ phase, which suggested Kank1 as a mediator of cell proliferation and apoptosis [23,24]. Over the last five years, accumulating evidence has confirmed that Kank1 plays a tumor-suppressive role in many malignancies [25,26,27,28,34,35]. Likewise, Kank1 depletion resulted in centrosomal amplification, and Kank1 overexpression inhibited the Wnt3a-stimulated F-actin formation [22]. Moreover, Kank1 has been reported to promote apoptosis via the mitochondrial pathway and the YAP/TAZ pathway [25,26,36]. These previous studies suggest that Kank1 plays a significant role in the inhibition of cell proliferation by arresting the cell cycle and promoting proapoptotic pathways. In the present study, Kank1 knockdown increased F-actin formation and myoblast proliferation and promoted cell cycle progression in C2C12 cells. Therefore, we suggest that Kank1 regulates myogenic differentiation by inhibiting proliferation and arresting the cell cycle of myoblasts during differentiation.

This study also showed that Kank1 knockdown induced F-actin accumulation in C2C12 myoblasts. Several studies have helped to establish our understanding of the roles of Kank1 in F-actin dynamics and focal adhesion [18,22,24,32,37]. Kank1 has been suggested to negatively control cytoskeleton remodeling in the cytoplasm by inhibiting actin polymerization [20,21,31,32,38]. The Rho family of GTPases (e.g., Rac, Rho, and Cdc42) serve as molecular switches and regulate various signal transduction pathways, especially those associated with stress fiber formation and membrane ruffling [21,22,32]. Kank1 can regulate actin polymerization negatively by inhibiting the binding between Rac1 and IRSp53 [21]. Furthermore, Kank1 could inhibit the activation of RhoA in a 14-3-3 protein-mediated manner [32]. On the other hand, the overexpression of Kank1 reduced actin polymerization and diminished actin stress fiber formation in NIH3T3 cells [22]. Therefore, these recent studies imply that F-actin accumulation observed in Kank1-depleted C2C12 myoblasts might be due to the inhibitions of Rho GTPases, such as RhoA and Rac1.

How then does Kank1 regulate myoblast proliferation and differentiation at the molecular level? Interestingly, the actin cytoskeleton has been proposed to be a crucial regulator of YAP1 [39], and the accumulation of F-actin in myoblasts has been reported to impair myogenic differentiation [14,40]. YAP1 phosphorylation by Hippo kinase enhances the proteasomal breakdown of YAP1, which inhibits its nuclear translocation [41]. Furthermore, F-actin accumulation has been reported to inhibit the Hippo signaling pathway and result in the nuclear translocation of YAP1 [30]. Our results show that Kank1 knockdown in myoblasts led to F-actin accumulation but reduced the phosphorylation of cytoplasmic YAP1 and, thus, facilitated the nuclear localization of YAP1 (Figure 2). Since YAP1 is a transcriptional co-activator that modulates cell proliferation, organ growth, and survival, YAP1-mediated mechanotransduction has also been proposed to enhance cell proliferation and cell cycle progression [10,42]. Furthermore, F-actin accumulation has been shown to promote cell division, growth, and proliferation by increasing the nuclear localization of YAP1 [43,44]. We found that Kank1 knockdown induced the expressions of the YAP1 target genes CCNB1 and CCND1, which are essential for cell cycle progression and proliferation. This finding is reminiscent of our previous observations that depletion of cofilin-2 (CFL2, an F-actin depolymerizing protein) increased F-actin accumulation, facilitated cell cycle progression, and promoted myoblast proliferation [14]. Moreover, knockdown of the F-actin capping and severing proteins (i.e., CapZ and Gelsolin) also resulted in the nuclear translocation of YAP1 and cell proliferation via a mechanotransduction mechanism [11,45]. Thus, our findings suggest that Kank1 affects myoblast proliferation and cell cycle progression by modulating F-actin and YAP1 in the Hippo signaling pathway. Nevertheless, the relationship between Kank1 and YAP1 and their roles in skeletal myogenesis has not been reported in animal models. Moreover, there is still controversy over the function of YAP1 in skeletal muscle. Previously, overexpression of YAP1 in skeletal muscle was found to induce muscle hypertrophy via mTOR pathways and interaction with TEAD [46,47]. In contrast, a transgenic knock-in mouse with constitutively active YAP1 in skeletal muscle exhibited muscle atrophy and myopathy [48]. Thus, further research is necessary to investigate how Kank1/YAP1 regulates skeletal muscle fibers in vivo.

## 5. Conclusions

In summary, this study revealed the essential role played by Kank1 in actin remodeling, cell proliferation, and myogenic differentiation of progenitor cells. Notably, the expression of Kank1 was altered during the myoblast differentiation, and Kank1 depletion drastically impeded the myogenic differentiation of myoblasts. Furthermore, we demonstrated that Kank1 is required to regulate myoblast proliferation and cell cycle progression properly. Kank1 knockdown augmented F-actin and enhanced the nuclear localization of YAP1, thereby promoting myoblast proliferation and hindering myogenic regulatory factor expressions and differentiation. Thus, Kank1 is essential for myogenic differentiation and myotube formation via F-actin/YAP1 axis regulation.

## Data Availability

The data presented in this study are available on request from the corresponding author.

## Supplementary Materials

The following are available online at https://www.mdpi.com/article/10.3390/cells11132030/s1, Supplementary data: Table S1: Oligonucleotide sequences for transfection, Table S2: Primer lists and conditions for *q*RT-PCR, Table S3: Antibodies list.
