# Supplementary material for: Kank1 Is Essential for Myogenic Differentiation by Regulating Actin Remodeling and Cell Proliferation in C2C12 Progenitor Cells
Source: Cells. 2022 Jun 26;11(13):2030. doi: 10.3390/cells11132030 (PMC9265739; doi:10.3390/cells11132030)
Supplement: Supplementary file 1 [file cells-11-02030-s001.zip › cells-1748288-supplementary.pdf]

Table S1. Oligonucleotide sequences for transfection

Gene	Primer sequence (5´-3´)
scRNA (control RNA)	UCACAACCUCCUAGAAAGAGUAGA
Kank1 siRNA (siKank1)	CAGAGAAGGACAUGCAGGUUU

Table S2. Mouse primer lists and conditions for *q*RT-PCR

Gene		Primer sequence (5´-3´)	Product size	Annealing Temp (°C)	Concentration		Cycle
					cDNA	Primer	
GAPDH	F.P	AACATCAAATGGGGTGAGGCC	252	58	2 ng/µl	0.5 µM	40
	R.P	GTTGTCATGGATGACCTTGGC					
CCNB1	F.P	GAGCTATCCTCATTGACTGG	125	58			
	R.P	CATCTTCTTGGGCACACAAC					
CCND1	F.P	ACCAATCTCCTCAACGACCG	228	58			
	R.P	ACGGAAGGGAAGAGAAGGG					

Table S3. Antibodies list

Antibody	Type	Targeted species	Manufacturer	Cat. No.	Dilution ratio*
Kank1	Monoclonal	Mouse	Santa Cruz Biotechnology, Dallas, TX, USA	sc-517629	1:5,000
MyHC	Monoclonal	Mouse	DSHB, Iowa, IA, USA	MF20	1:1,000
MyoD	Monoclonal	Mouse	Santa Cruz Biotechnology, Dallas, TX, USA	sc-377460	1:1,000
MyoG	Monoclonal	Mouse	Santa Cruz Biotechnology, Dallas, TX, USA	sc-12732	1:1,000
YAP1	Monoclonal	Rabbit	Cell Signaling Technology, Danvers, MA, USA	14074S	1:10,000
pYAP1	Polyclonal	Rabbit	Cell Signaling Technology, Danvers, MA, USA	4911S	1:10,000
Lamin B2	Monoclonal	Rabbit	Abcam, Cambridge, United Kingdom	ab151735	1:2,500
α-Tubulin	Monoclonal	Mouse	DSHB, Iowa City, IA, USA	12G10	1:2,000
β-Actin	Monoclonal	Rabbit	Sigma-Aldrich Chemical, St. Louis, USA	A2066	1:10,000
Antibodies HRP-linked anti-rabbit IgG			Cell Signaling Technology, Danvers, MA, USA	#7074	1:10,000
Goat anti-mouse(H+L)			Invitrogen, Thermofisher Scientific, Waltham, MA, USA	#32430	1:2,000

*All blots were visualized using a Femto reagent (Thermofisher Scientific).
